# TNFα Levels and Macrophages Expression Reflect an Inflammatory Potential of Trigeminal Ganglia in a Mouse Model of Familial Hemiplegic Migraine

**DOI:** 10.1371/journal.pone.0052394

**Published:** 2013-01-11

**Authors:** Alessia Franceschini, Sandra Vilotti, Michel D. Ferrari, Arn M. J. M. van den Maagdenberg, Andrea Nistri, Elsa Fabbretti

**Affiliations:** 1 Neuroscience Department, International School for Advanced Studies, Trieste, Italy; 2 Department of Neurology, Leiden University Medical Centre, Leiden, The Netherlands; 3 Department of Human Genetics, Leiden Genetics University Medical Centre, Leiden, The Netherlands; 4 Center for Biomedical Sciences and Engineering, University of Nova Gorica, Nova Gorica, Slovenia; Charité-University Medicine Berlin, Germany

## Abstract

Latent changes in trigeminal ganglion structure and function resembling inflammatory conditions may predispose to acute attacks of migraine pain. Here, we investigated whether, in trigeminal sensory ganglia, cytokines such as TNFα might contribute to a local inflammatory phenotype of a transgenic knock-in (KI) mouse model of familial hemiplegic migraine type-1 (FHM-1). To this end, macrophage occurrence and cytokine expression in trigeminal ganglia were compared between wild type (WT) and R192Q mutant Ca_V_2.1 Ca^2+^ channel (R192Q KI) mice, a genetic model of FHM-1. Cellular and molecular characterization was performed using a combination of confocal immunohistochemistry and cytokine assays. With respect to WT, R192Q KI trigeminal ganglia were enriched in activated macrophages as suggested by their morphology and immunoreactivity to the markers Iba1, CD11b, and ED1. R192Q KI trigeminal ganglia constitutively expressed higher mRNA levels of IL1β, IL6, IL10 and TNFα cytokines and the MCP-1 chemokine. Consistent with the report that TNFα is a major factor to sensitize trigeminal ganglia, we observed that, following an inflammatory reaction evoked by LPS injection, TNFα expression and macrophage occurrence were significantly higher in R192Q KI ganglia with respect to WT ganglia. Our data suggest that, in KI trigeminal ganglia, the complex cellular and molecular environment could support a new tissue phenotype compatible with a neuroinflammatory profile. We propose that, in FHM patients, this condition might contribute to trigeminal pain pathophysiology through release of soluble mediators, including TNFα, that may modulate the crosstalk between sensory neurons and resident glia, underlying the process of neuronal sensitisation.

## Introduction

Familial Hemiplegic Migraine type 1 (FHM-1) is a rare monogenic subtype of migraine caused by missense mutations in the *CACNA1A* gene, which encodes the α1 subunit of Ca_V_2.1 (P/Q-type) Ca^2+^ channels [1], [2]. A transgenic knock-in (KI) FHM mouse model generated by introducing the human pathogenic FHM-1 mutation R192Q into the endogenous *Cacna1a* gene [3], can be used to better understand the underlying pathophysiology [4], [5]. The R192Q KI mice show increased neuronal Ca^2+^ influx and enhanced glutamate release [6], which can explain the increased susceptibility to cortical spreading depression [3], [6], the underlying mechanism of the migraine aura [7] and perhaps the headache mechanisms [8], [9]. It is noteworthy that, in R192Q KI mice, hyperactivation of trigeminal ganglion ATP-sensitive nociceptors [10] may represent an important process for sensitization of sensory neurons that trigger headache. As it has been suggested that FHM and common migraine may share some pathogenetic mechanisms [11], [12], [13], the study of FHM mechanisms may, thus, provide unique insights into the pathophysiology of migraine [11] and familial hemiplegic migraine will continue to be one of the main models to study molecular genetics of migraine [13].

The etiology of migraine pain remains poorly understood because the exact cause of headache onset, and its predisposing factors, remains a matter for investigation. One theory proposes that several migraine mediators including CGRP and ATP [14], [15] released by sensory neurons and satellite cells concur to trigger hyperactivity of the peripheral afferents of trigeminal sensory neurons innervating the dura mater [16]. Another possibility is that, during a migraine attack, activated macrophages and other non-neuronal cells might induce a meningeal “sterile inflammation” [17], [18], a phenomenon that can contribute to strong headache when associated with local production of inflammatory substances [9], [19]. Furthermore, it has been reported that inflammation in the broad trigeminal nerve territory is often observed during migraine attacks [20] so that acute administration of corticosteroids has been tested to block pain, albeit with mixed results [21]. It is, however, unclear how common these mechanisms are to the various migraine subtypes.

Since the functional cross talk between non-neuronal cells and sensory neurons seems an important phenomenon in the pathophysiology of chronic pain [22], we investigated whether inflammatory-like alterations are present in the trigeminal ganglia of R192Q KI and WT mice, as sensory neuron somata integrate (via ionic conductances) afferent nociceptive inputs and send frequency-coded signals to trigeminal brainstem nuclei [23], [24].

We, therefore, examined, in trigeminal ganglia of R192Q KI and WT mice, the presence, morphology and distribution of macrophages. Furthermore, we investigated trigeminal ganglion ability to express cytokines typical of inflammatory responses under basal conditions and following a strong inflammatory stimulus evoked by the endotoxin lipopolysaccaride (LPS; [25]). While LPS is not a model tool to induce migraine pain, it can induce expression and release of TNFα, which is known to have a role in trigeminal ganglia sensitisation [26], [27], [28]. Our data suggest that R192Q KI trigeminal ganglia expressed a cellular and molecular phenotype that responded more readily to inflammatory stimulation. These alterations outline potential mechanisms that might facilitate trigeminal ganglion excitability through the complex crosstalk between neurons and glia that occurs during pain generation [22], [29].

## Results

### Comparison of macrophages in trigeminal ganglia from WT and R192Q KI mice

**Figure 1:**
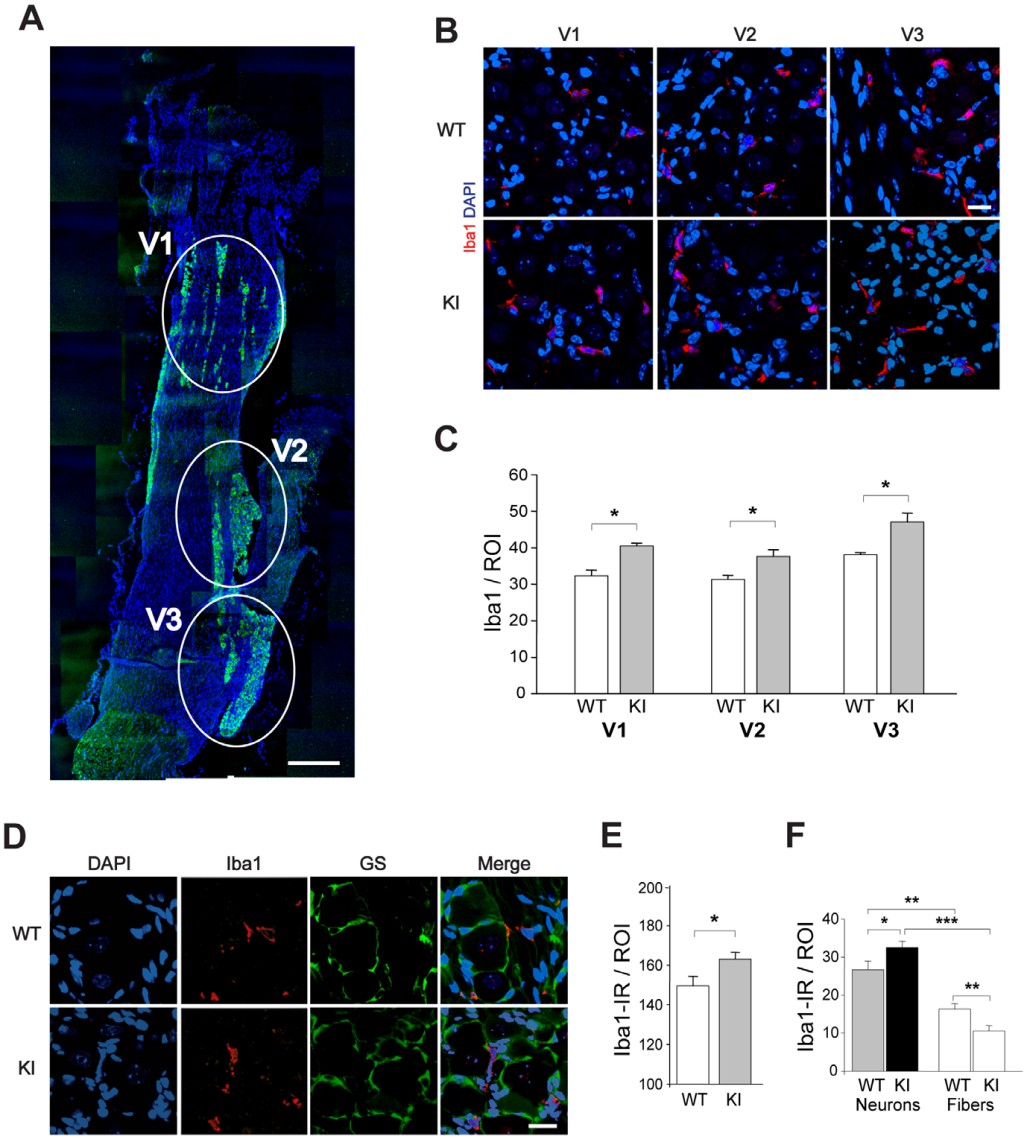
Iba1 immunoreactivity in trigeminal ganglia from in WT and R192Q KI mice. *A*, Representative confocal microscopy image of a longitudinal section of mouse trigeminal ganglion immunostained for neuronal β-tubulin III (green), and labeled with DAPI (blue). Discrete distribution of neuronal somata in three histological subdivisions of the trigeminal ganglion (i.e. V1, V2 and V3) is indicated by ellipsoids. Scale bar: 300 µm. *B*, Representative confocal microscopy images of WT (top row) or R192Q KI (bottom row) trigeminal ganglion sections from different V1, V2 and V3 regions of WT and KI ganglia and immunostained with Iba1 (red). Nuclear signal is also shown (blue). Scale bar: 15 µm. *C*, Histograms quantify Iba1-positive cells in V1, V2 and V3 regions (ROI: 370 µm×370 µm) of WT and KI trigeminal ganglia. Data were collected in parallel from at least 3 WT and 3 KI mice; * *p*<0.01. *D*, Panels show confocal images of WT (top row) and R192Q KI (bottom row) trigeminal ganglion sections immunostained with anti-Iba1 (red, macrophages) and the anti-glutamine synthetase antibodies (GS, green, satellite glial cells). Nuclei were labeled with DAPI (blue). Note the peculiar GS-positive immunostaining of satellite glial cells typically surrounding neurons (green). No Iba1 co-localisation was found in GS-immunolabeled cells. Scale bar: 20 µm. *E*, Histograms quantify the Iba1-immunoreactivity (IR) in whole ganglion slices from R192Q KI with respect to WT samples. Data are from an average of 30 ROIs per whole ganglion, respectively from *n* = 6 WT and KI mice. ROI: 640×480 µm. n = 6 mice, * *p*<0.001. *F*, Iba1 immunoreactivity was counted in neuronal- (β-tubulin III-positive) or fiber- (MAP-2-positive, not shown) enriched ganglia regions; *n* = 3 mice; * *p*<0.05; ** *p*<0.01; *** *p*<0.001.

Fig. 1 A shows the typical subdivision of trigeminal ganglion neurons (immunostained for β-tubulin III) in three clusters that correspond to the V1, V2 and V3 regions [30]. Inspection (at higher magnification) of the three subdivisions (as exemplified in Fig. 1 B) indicated sparse occurrence of Iba1-positive cells that were more abundant in R192Q KI sections throughout (Fig. 1 C). In peripheral tissues, Iba1-positive cells are usually identified as macrophages [31], [32]. In line with the notion that Ca_V_2.1 (P/Q-type) calcium channels are exclusively expressed by neurons [12], we confirmed that no Ca_V_2.1 mRNA signal was detected in cultured peritoneal macrophages (n = 5 mice). In trigeminal ganglia, Iba1-immunoreactive cells were distinct from satellite glial cells, as demonstrated by the lack of co-localization of Iba1 signal with the glutamine synthetase signal (GS; Fig. 1 D), a canonical satellite cell marker [33]. Across a broad ganglion area, Iba1-positive cells were not only more frequent in KI ganglia (Fig. 1 E), but they were also preferentially localized in the proximity of neuronal cell bodies rather than fibers (Fig. 1 F).

**Figure 2:**
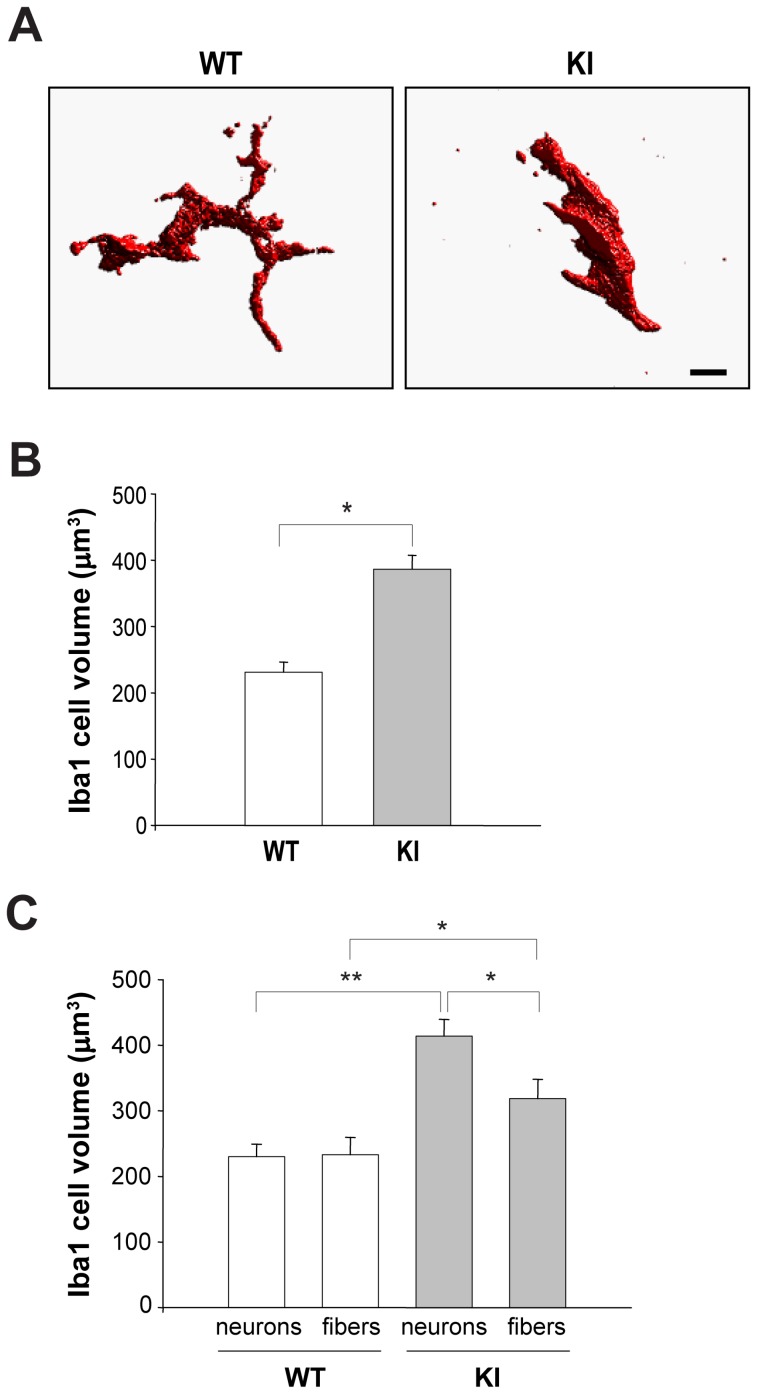
Different morphology of Iba1 immunoreactive cells in WT and R192Q KI trigeminal ganglia. *A*, Examples of 3D reconstruction of Iba1-positive cells (from confocal Z-stacks, each 0.5 µm-tick) from WT or R192Q KI ganglia. Note large branching of WT cell vs compact, process-free KI cell morphology. Scale bar: 5 µm. *B*, Histograms quantify average volume (µm^3^) of Iba1-positive cells in WT and R192Q KI ganglia, obtained from voxel analysis of 3D images. Data were collected from three independent experiments with a total of 83 cells for WT and 70 cells for R192Q KI; * *p*<0.001. *C*, Histograms quantify the average volume of Iba1-positive cells from different neurons or fibers enriched areas of WT and R192Q KI ganglia; *n* = 20–50 cells (3 WT and 3 KI mice). * *p*<0.05; ** *p*<0.01.

Iba1 expression is typically upregulated in activated macrophages/microglia that exhibit distinct morphology with ameboid shape and short processes resulting in a larger cell volume [34], [35], [36]. 3D reconstruction of confocal images of WT and R192Q KI trigeminal ganglion Iba1-positive cells showed that KI macrophages displayed increased cell volume (with amoeboid morphology and shorter processes) compared with WT cells (Fig. 2 A, B). Interestingly, larger macrophages were detected near KI ganglion neurons (Fig. 2 C).

These data suggest that in R192Q KI ganglia Iba1 macrophages showed an activation state with their preferential location close to neuronal somata.

### Biomarker characteristics of Iba1 cells in WT or R192Q KI trigeminal ganglia

**Figure 3:**
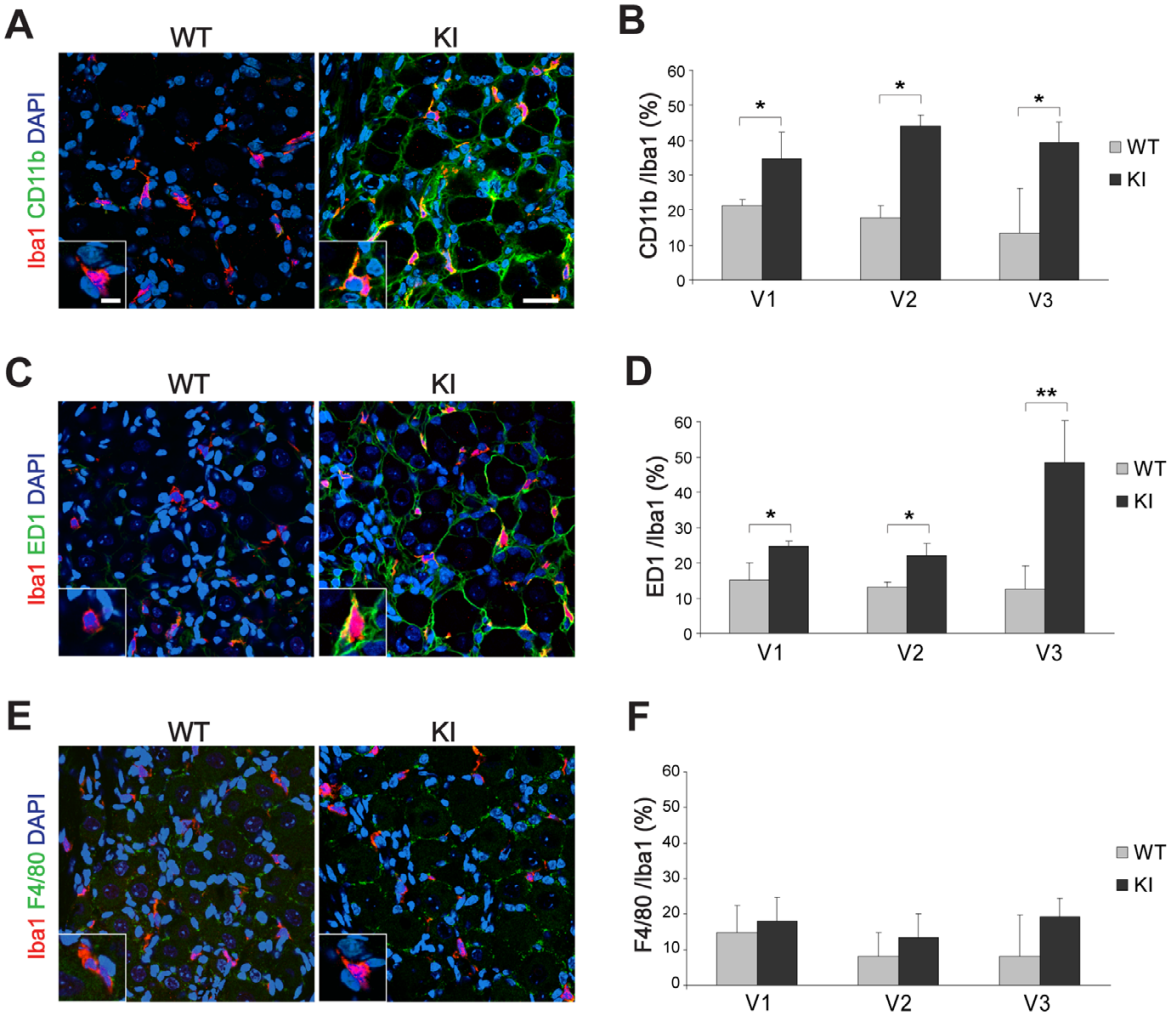
Characterization of Iba1-positive cells in trigeminal ganglia from WT and R192Q KI mice. *A, C, E*, Representative confocal images of WT or R192Q KI trigeminal ganglion sections (from V3 region) immunostained for Iba1 (red) and CD11b (*A*), ED1 (*C*), or F4/80 (*E*) in green. Nuclei were labeled with DAPI (blue). Scale bar: 40 µm. Insets represent larger magnification of immunoreactive Iba1 cells. Scale bar: 7 µm. *B, D, F,* Histograms quantify the percentage of occurrence of Iba1 signal with CD11b (*B*), ED1 (*D*) or F4/80 (*F*) in different WT and KI ganglion regions (V1, V2 or V3). Expression of different markers was quantified in Iba1 positive cells only. *n* = 3 WT and 3 KI mice; * *p*<0.05; ** *p*<0.01. Data are expressed as mean ± S.D.

Fig. 3 A shows examples of confocal microscopy images from WT or R192Q KI ganglion sections that were co-immunostained with antibodies against Iba1 and CD11b, an adhesion molecule marker for active macrophages and microglia [37], [38], [39].

Expression of CD11b was quantified in Iba1-positive cells only. In WT ganglia, 20% of Iba1-positive cells (without topographical difference within the three areas) expressed CD11b (Fig. 3 B). On the other hand, in KI tissue, the percentage of Iba1 and CD11b co-expressing cells was significantly higher (up to 45% of Iba1-positive cells; Fig. 3 B).

Iba1-negative satellite cells (see Fig. 1 D) were also stained with the CD11b antibody in R192Q KI sections (Fig. 3 A, right panel). Nevertheless, in KI ganglia, co-localization of CD11b and Iba1 in macrophages was clearly confirmed in fiber regions devoid of satellite cells (Figure S1).

Similar experiments were performed to evaluate the presence of the macrophage antigen ED1 (CD68), a glycoprotein highly expressed by monocytes and tissue macrophages and associated with larger phagocytic ability [40] (Fig. 3 C, D). While, in WT tissue, ED1/Iba1 double-positive cells were fewer than 15%, this value was significantly higher in R192Q KI ganglia, in particular in the V3 region (Fig. 3 D). ED1 was also detected in KI satellite glial cells (Fig. 3 C, right).

Expression of the F4/80 antigen is restricted to most resident mature macrophages and quiescent microglia [41], [42], [43], and has recently been linked to the induction of immunological tolerance [44]. In WT and R192Q KI ganglia, the F4/80 signal was rarely observed in Iba1-positive cells and not significantly different between WT and R192Q KI ganglia (Fig. 3 E, F).

These data indicate different subpopulations of Iba1-positive cells of R192Q KI ganglia.

### Cytokine expression in trigeminal ganglia from WT and R192Q KI mice

**Figure 4:**
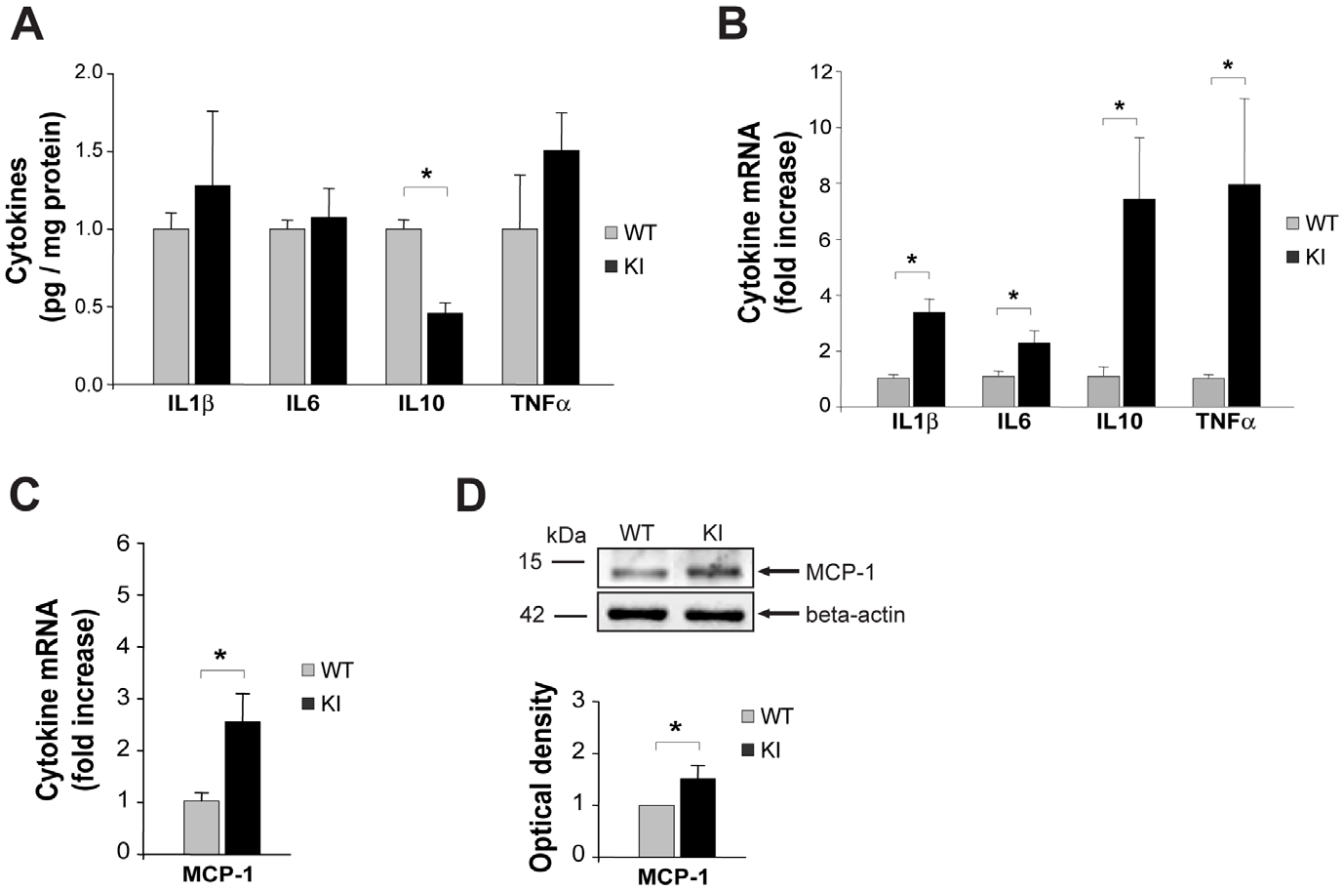
Expression of inflammatory mediators in WT and R192Q KI trigeminal ganglia under basal conditions. *A*, Histograms quantify IL1β, IL6, IL10 and TNFα cytokine protein levels from WT or R192Q KI ganglia; *n* = 4 WT and 4 KI mice; data were normalized on total protein content, and represented as fraction of WT. * *p*<0.001. *B*, Real-time RT-PCR experiments quantify IL1β, IL6, IL10 and TNFα mRNA levels in WT and R192Q KI trigeminal ganglia. PCR data were normalized with respect to corresponding GAPDH and β-Tubulin housekeeping gene expression and expressed as fraction of WT; *n* = 4 WT and 4 KI mice; * *p*<0.05. *C*, Real-time RT-PCR experiments quantify MCP-1 mRNA levels in WT and R192Q KI trigeminal ganglia (expressed as in *B*); *n* = 4 WT and 4 KI mice; * *p*<0.05. *D*, Representative western blot experiment of WT or R192Q KI trigeminal ganglia extracts immuno-probed with anti-MCP-1 antibodies. Actin levels were used as loading control. Histograms quantify the differences. *n* = 3; * *p*<0.05.

A neuroinflammatory role of higher cytokine and chemokine expression is thought to be associated with chronic pain models [29]. We measured the protein and RNA cytokines content of whole WT and R192Q KI ganglion extracts (Fig. 4 A, B). ELISA experiments demonstrated no significant difference in protein levels of IL1β, IL6 and TNFα between WT or R192Q KI lysates. The anti-inflammatory cytokine IL10, however, was significantly lower in R192Q KI than WT ganglia (Fig. 4 A). Although these observations, at the protein level, do not suggest an on-going inflammatory state in R192Q KI ganglia, it is interesting that cytokines mRNA data were, however, significantly higher in R192Q KI than WT samples for all four genes (Fig. 4B).

High levels of monocyte chemoattractant protein-1 (MCP-1; also known as chemokine receptor type 2, CCR2) are associated with meningeal neuroinflammation in migraine [45]. As shown in Fig. 4 C, elevated mRNA levels of this marker were found in KI rather than WT ganglia; furthermore, stronger expression of the MCP-1 protein by KI lysates was observed (Fig. 4 D), consistent with the more numerous DAPI-positive elements (4330±60 for KI vs 3740±70 for WT; *p*<0.001; *n* = 4), and suggestive of possible cell recruitment to trigeminal ganglia of R192Q KI mice.

**Figure 5:**
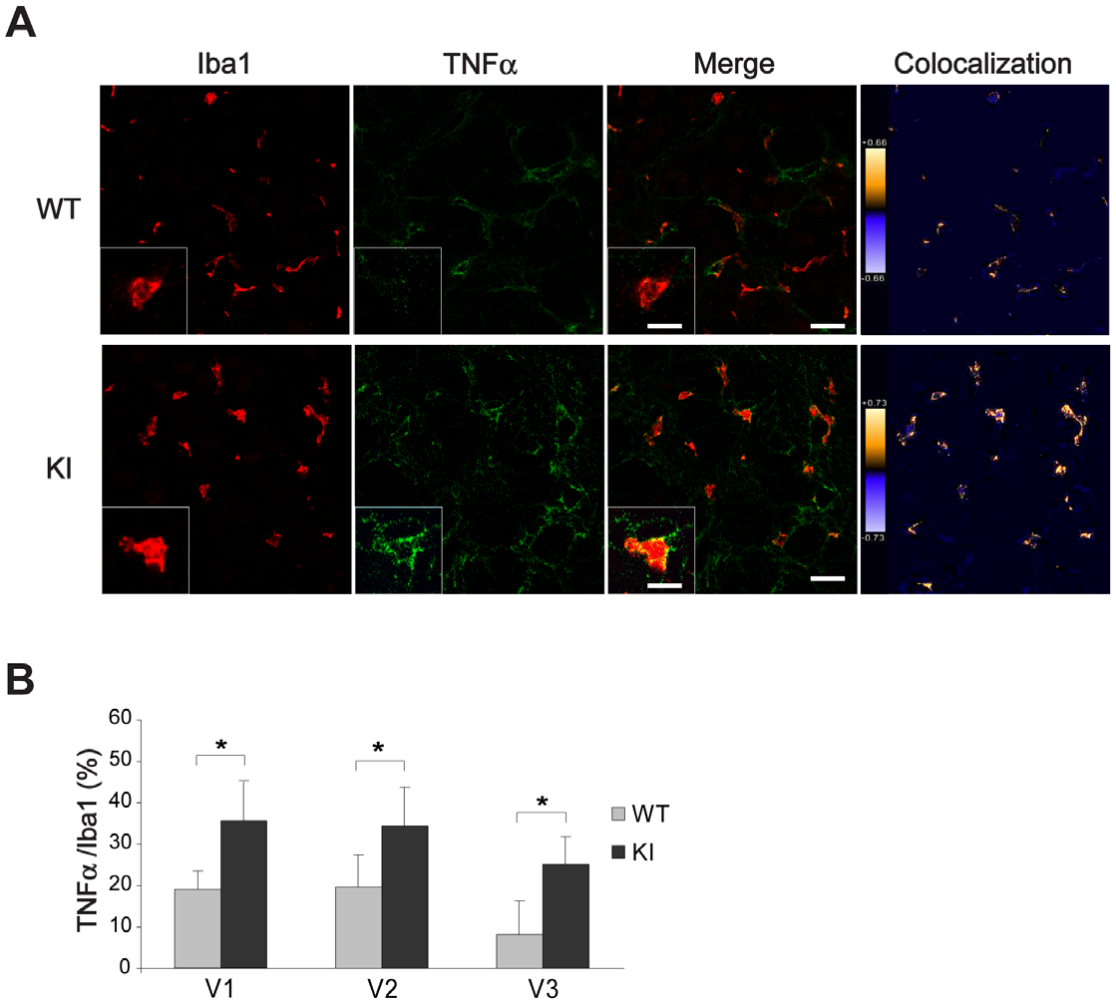
TNFα expression in WT and KI ganglia. *A*, Representative confocal microscopy images of WT (top row) or R192Q KI (bottom row) trigeminal ganglion sections immunostained for Iba1 (red) or TNFα(green) in basal condition. Pseudocolor images showing areas of high (yellow) and low (blue) Iba1-TNFα expressing cell co-localization. Color scale was also included. Note TNFα immunostaining detected as spots along perimembrane regions. The larger magnification insets show immunostaining of Iba1-TNFα signal (yellow) in KI rather than WT. Scale bar: 30 µm, for large images; Scale bar: 10 µm for larger magnification insets. *B*, Histograms quantify the percentage of TNFα immunoreactivity over the total of Iba1 expressing cells in different V1, V2 or V3 trigeminal regions (ROI: 370×370 µm). *n* = 4 WT and 4 R192Q KI mice; * *p*<0.05. Data are expressed as mean ± S.D.

Since TNFα powerfully sensitizes trigeminal ganglia [27], [28], we studied the potential difference in TNFα expression at the single cell level between WT and R192Q KI ganglia. Fig. 5 A exemplifies confocal microscopy images from WT or R192Q KI ganglia co-stained with antibodies against Iba1 and TNFα in WT and KI ganglia. In R192Q KI ganglia, the number of Iba1-positive cells that were also immunoreactive for TNFα was significantly higher (Fig. 5 A, B), supporting the hypothesis of a stronger inflammatory potential of non-neuronal cells in KI mouse ganglia with respect to WT.

### LPS-evoked changes in TNFα expression

**Figure 6:**
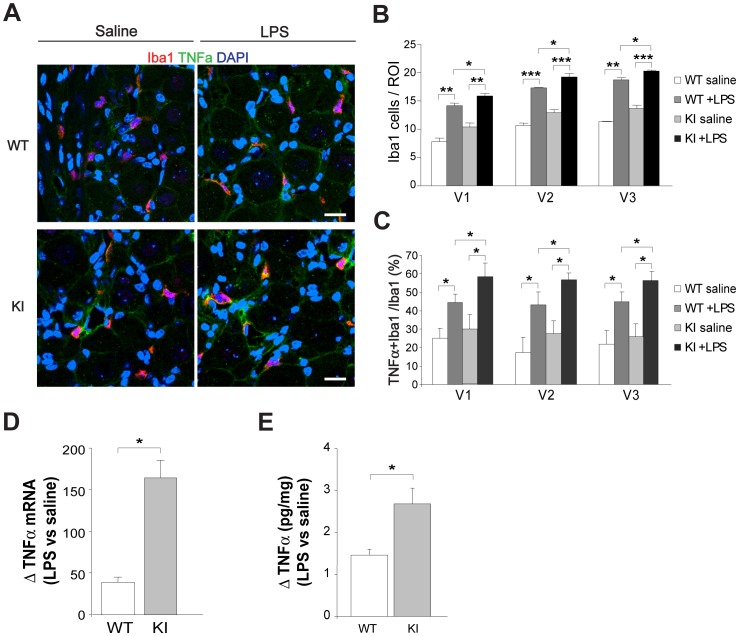
LPS evoked acute TNFα expression in R192Q KI ganglia. *A*, Representative confocal microscopy images of WT (top row) or KI (bottom row) trigeminal ganglion sections immunostained for Iba1 (red) or TNFα(green) after saline (left) or LPS injection (i.p., 5 h; right). LPS evokes TNFα expression in WT and KI after injection. Scale bar: 20 µm. *B, C,* Histograms quantify the occurrence of Iba1 signal (*B*) and Iba1-TNFα co-localisation (*C*) in different V1, V2 or V3 trigeminal ganglion regions from WT or KI mice, after saline or LPS injection (i.p., 5 h). ROI: 370×370 µm. *n* = 3 WT and 3 R192Q KI mice; * *p*<0.05; ** *p*<0.01; *** *p*<0.001. *D*, Histograms quantify changes in TNFα mRNA fold increase in WT or R192Q KI ganglia following LPS-injection (i.p., 1 h). Data are expressed as fold increase with respect to saline-injected mice samples; *n* = 3 WT and 3 KI mice; * *p*<0.05. *E*, TNFα protein levels in whole ganglia (pg cytokine/mg protein content) from LPS-injected (i.p. 5 h) WT or R192Q KI mice, expressed as fold increase with respect to saline-injected mice. *n* = 3 WT and 3 KI mice; * *p*<0.05.

To further test trigeminal ganglion reactivity, we used a standard inflammatory trigger such as LPS, as a tool to study differential inflammatory-evoked responses in WT and R192Q KI mice. Even if LPS cannot be considered a migraine-provoking tool, it is often used to investigate microglia activation [25], [46], as its intraperitoneal (i.p.) injection induces an early (1 h) rise in TNFα mRNA followed by strong expression of TNFα protein by macrophages a few h later [46]. In agreement with data from DRG ganglia [45], 5 h after LPS injection, there was a significant increment in TNFα immunostaining (Fig. 6 A) and a larger number of Iba1-positive cells in the three areas of R192Q KI ganglia with respect to those in WT mice (Fig. 6 A, B). Furthermore, we observed a significantly higher occurrence of TNFα and Iba1 co-staining (Fig. 6 C). One h after LPS injection, the increase in TNFα mRNA levels was more pronounced in R192Q KI compared with WT ganglion lysates (Fig. 6 D). This phenomenon was followed by a delayed (5 h) increment in TNFα protein expression detected with ELISA assay (Fig. 6 E) occurring more strongly in KI than WT ganglia.

These results suggest an enhanced response of R192Q KI ganglia to a standard inflammatory stimulus.

## Discussion

The principal finding of our study is that trigeminal ganglia of a transgenic mouse model of FHM-1, a rare subtype of inherited migraine, exhibited a new basal phenotype of stronger reactivity evidenced by macrophage morphology changes and an abnormal cytokine and chemokine expression profile.

### Can abnormalities in R192Q KI ganglia help to understand the basic mechanisms of migraine pain?

The trigeminal phenotype of R192Q KI mice remains to be fully clarified. It is, therefore, relevant to investigate changes in molecular and cellular mechanisms in the trigeminal ganglion which is responsible for conveying nociceptive signals to the brainstem.

Migraine is a complex disease with pleiotropic symptomatology, amongst which strong, long-lasting headache is the major unifying complaint. FHM-1 presents certain analogies and differences with common migraine as extensively reviewed by Russell and Ducros [13]. Thus, FHM-1 patients typically experience a stronger aura syndrome often lasting longer than in common migraine, and comprising extensive motor and sensory dysfunction. These data suggest that the mechanisms leading to the initiation of cortical spreading depression might differ between FHM-1 and the most common forms of migraine [13]. In particular, FHM-1 patients are not hypersensitive to CGRP [47] and nitric oxide [48], by contrast with patients with migraine with or without aura [49]. Since administration of CGRP or of a nitric oxide donor does not induce migraine in healthy subjects [50] it is likely that the hypersensitivity of migraineurs to these substances implies abnormal signaling downstream of the primary mechanism of CGRP and nitric oxide activities.

Because there is a large family of soluble factors (migraine mediators) that can trigger and perpetuate migraine [14], it might be possible to hypothesize that FHM-1 migraine is supported by other substances besides CGRP and nitric oxide. In line with this possibility is the report of apparently lower immunocytochemical expression of CGRP in R192Q KI ganglia [51] together with higher CGRP release [15], [19], suggesting misdirected signaling of CGRP probably toward non-neuronal cells.

Notwithstanding the complexity of this field, it is clear that the headache characteristics in FHM-1 are usually those of a typical attack of migraine [13] since, apart from the motor weakness or hemiparesis during aura, FHM attacks resemble migraine with aura attacks [52]. As local inflammation has been proposed to support migraine pain [17], [18], [19], [20], we sought to investigate if a special phenotype of trigeminal ganglia (responsible for conveying nociceptive signals to the brainstem) consistent with propensity to neuroinflammation might be observed.

### Experimental neuroinflammation within trigeminal ganglia

The molecular and cellular phenotype characteristics of R192Q KI mice could perhaps reside in a different basal condition with distinct contributions by various cells to the process of neuronal sensitization. Studies of meningeal tissue from migraine animal models and migraineurs show molecular and histological changes typical of neuroinflammation [8], [53], [54], [55]. In particular, experimental studies of rat dura mater have indicated that a strong chemical trigger like, for example glyceryl trinitrate or LPS, can induce expression of several cytokines such as IL1β, IL6 and the nitric oxide synthetic enzyme iNOS [56]. Release of soluble factors including inflammatory cytokines is proposed to activate small afferent fibers to produce headache and to further stimulate release of neuropeptides from neuronal afferents [57]. It is, however, unclear whether trigeminal ganglia per se are also affected by these neuroinflammatory processes, and how much their phenotype may be generalized to other migraine types. This possibility is not unlikely because antigen-presenting immune cells have been characterized in human trigeminal ganglia [58] and certain chronic pain models are associated with neuroinflammatory changes in dorsal root ganglia [59]. Our data support that, at the level of trigeminal ganglia, a FHM-1 mouse model with a R192Q missense mutation in the α1 subunit of voltage-gated Ca_V_2.1 calcium channels elicits cellular and molecular changes consistent with this hypothesis. Of course, since these channels are typical of neurons [12], and macrophages did not express Ca_V_2.1 channels, any observed molecular and/or cellular alteration within ganglion cell populations implies cross talk between neurons and non-neuronal cells.

### Macrophages in trigeminal ganglia of R192Q KI mice show activation state

Macrophages play an important role in tissue pathophysiological responses like chronic pain [29]. In trigeminal ganglia, Iba1-positive macrophages were a small cell population that, nonetheless, in R192Q KI ganglia, showed significant signs of activation (ameboid shape due to their larger volume [60], [61]) that resembled the one observed in sensory ganglia of injured rodents [62]. Future studies will be necessary to find out if macrophage changes can also be observed in dorsal root ganglia of R192Q KI mice.

Our study found no difference in macrophage intra-ganglion sub-distribution in relation to the three main branches of the trigeminal nerve. Consistent with these findings, it has been reported that, despite the few meningeal afferents to trigeminal ganglia observed with retrograde labeling [15], a chemical stimulation related to a specific topographic neuronal area causes alteration also in the other areas of the trigeminal ganglion [30]. Furthermore, functional studies have indicated widespread involvement of different ganglion regions, since Ca_V_2.1 expression is not restricted to the fine meningeal afferents of trigeminal ganglia only [15]. The relevance of our finding is not contradicting the notion that headache is the principal trigeminal pain in migraine. Nonetheless, an extensive clinical study carried out on 1,413 patients has indicated that, during an attack of migraine, the majority of them suffer from allodynia affecting the entire trigeminal territory [63], a finding confirmed to be independent from the presence of aura [64].

To characterise Iba1-positive macrophages in R192Q KI trigeminal ganglia, we found that a significant fraction of them co-expressed CD11b and ED1, but not F4/80. Interestingly, satellite cells surrounding neuronal somata of R192Q KI ganglia also showed large expression of CD11b and ED1 markers. Satellite glial cells in sensory ganglia tightly envelop the neuronal cell body to form discrete anatomical units [33], and may express an immune-related function within human sensory ganglia [58]. We propose that, in R192Q KI mice, active Iba1-positive cells that are located close to the neuronal units in the ganglia, with likely release of soluble factors, could mediate *de novo* expression of CD11b and ED1 in satellite glial cells, even if their functional role remains to be investigated.

### Cytokine profile of R192Q KI ganglia

In neuroinflammatory processes, immune cell activity is typically associated with higher expression of chemokines, like MCP-1 [65], [66], and cytokines that are believed to be key contributors to chronic pain [22], [67]. In R192Q KI ganglia, new antigen expression and macrophage activation were associated to larger cytokine mRNA levels, without significant changes in corresponding proteins. Still, the anti-inflammatory cytokine IL10 was lower in KI ganglia, a result which would be in line with their propensity to a neuroinflammatory reaction [68]. An alternative possibility to be explored is that the inflammatory nociceptive activation in the ganglia could arise from hyperactivity of the trigeminal vascular system leading to increased cytokine expression at the meninges.

During inflammatory conditions, activated macrophages release TNFα that modulates immune responses and stimulates crosstalk between neurons and glia to facilitate pain [22], [27], [68]. Studies of rat dorsal root ganglia have demonstrated that TNFα is primarily synthesized by ED1-expressing macrophages [46]. It is known that pro-inflammatory cytokines are rapidly released to stimulate the arrival and activation of immune cells generating an inflammatory response [69]. Our study indicates that R192Q KI trigeminal ganglia had higher mRNA and protein levels of the macrophage-related chemokine MCP-1/CCL2 involved in macrophage recruitment/mobility and activation [70], [71] and also found raised in the plasma of migraine patients [72], [73].

Electrophysiological studies will be necessary to investigate how inflammatory mediators might change the operation of ligand- and voltage-activated channels of trigeminal sensory neurons, and, consequently, shape the firing properties of these cells. For example, after injury, TNFα enhances the sodium current of rat DRG neurons, lowers the spike threshold and promotes high frequency firing [74]. Future investigations will need to analyze if this cytokine may differentially affect spike firing by WT and R192Q KI trigeminal neurons.

### Inflammatory stimulus strongly enhanced TNFα production

LPS per se is not considered to be an agent capable of inducing an acute attack of migraine. We employed LPS to explore whether the basal inflammatory profile of KI ganglia could be expressed (by LPS administration) into a biochemical substrate compatible with a strong inflammatory reaction and production of soluble factors (e.g. TNFα) potentially promoting the release of migraine mediators. Thus, using this approach, the basal pro-inflammatory potential of R192Q KI ganglia was readily converted into enhanced inflammatory reactivity together with elevated TNFα expression.

The origin of the neuroinflammatory reaction strongly detected in R192Q KI ganglia remains uncertain as it could be initiated in the ganglion itself perhaps because of the altered milieu caused by low-threshold spreading depression occurring in these mice [2], [3]. The innate immune system is increasingly viewed to play an important role in mediating chronic pain [75], especially through the Toll-like receptor 4 (TLR4) that was found also involved in several sterile inflammation processes and activated by endogenous ligands [76]. TLR4 is the primary target of bacterial LPS [77], [78] and induces the expression of proinflammatory cytokines and chemokines [79].

In addition, an inflammatory nociceptive activation developing at the meninges might have driven the increase in cytokine expression in the ganglion. Since the basal inflammatory reactivity of R192Q KI trigeminal ganglia has also been observed in primary culture [80], this finding is compatible with the view that the KI ganglion had a constitutive neuroinflammatory profile.

LPS activates monocytes and macrophages to produce proinflammatory cytokines such as TNFα [81]. Inflammatory cytokines such as TNFα contribute to peripheral sensitization of nociceptor neurons [82], and in particular, in migraine patients, raised concentrations of TNFα have been reported in the jugular blood of patients 2 h after the onset of an attack [57]. We found that, at the ganglion level, a strong rise in TNFα production by R192Q KI ganglia after LPS together with substantial increment in the number of Iba1-positive cells co-expressing TNFα. These observations are consistent with hyper-reactivity of R192Q KI ganglia to inflammatory stimulation to which various non-neuronal cell types could contribute with their own synthesis and release of TNFα. In fact, studies of mRNA and Western blotting could not identify the source of cytokines: nonetheless, single cell immunohistochemistry indicated that TNFα was abundantly expressed by macrophages.

## Conclusions

Future experiments will be necessary to identify the molecular mechanisms linking the gain-of-function of mutated Ca_V_2.1 channels in R192Q sensory neurons to the inflammatory profile of the ganglion tissue, and how raised TNFα production and release are converted into over-activity of nociceptors of trigeminal sensory neurons as experimentally observed in the R192Q KI mouse [10]. It is, however, feasible to hypothesize that TNFα is a significant player in triggering trigeminal pain as it can release a host of algogenic substances like BDNF [26] and CGRP [28]. In the FHM-1 mouse model, the genetic mutation not only confers a sensitized pain receptor phenotype to a subclass of sensory neurons [10], [15], but it can also modify the trigeminal ganglion microenvironment that may predispose to chronic pathological conditions specific for a subset of migraine patients, as reported in the current study. Previous investigations have demonstrated that R192Q KI mice are very susceptible to strong cortical spreading depression, believed to be the equivalent of human aura [2], [3], [7]. An interesting possibility is that former bouts of cortical spreading depression with associated release of soluble factors into the extracellular space might have primed macrophages to synthesize and liberate proinflammatory mediators. Whether this process actually occurs in vivo during an aura remains a matter of conjecture. Nonetheless, our data add the role of inflammatory cells to the growing body of evidence showing how macrophages/microglia (via purinergic receptors) play a key role in pain signaling through neuron/glia interaction [83].

## Methods

### Animal procedures

Ca_V_2.1 R192Q KI and WT littermates (P30) were used. Animals were maintained in accordance with the guidelines of the Italian Animal Welfare Act and their use has been approved by the Local Ethical Committee. Our experimental protocols, have been approved by SISSA ethics committee board and by National Ministry of Health (reference # 13184), as they are in accordance with the European Union guidelines. Genotyping was performed by PCR as previously reported [3]. To evoke acute inflammation, WT or R192Q KI mice (P30) were injected intraperitoneally (i.p.) with a single dose of saline (sham) or LPS (5 mg/kg, from *E. coli* 0111:B4; Sigma, Milan, Italy) 1 or 5 h prior to sacrificing the animals [46]. Ganglion tissue samples were collected and processed in parallel for WT and R192Q KI mice.

### Immunohistochemistry

For immunohystochemistry, WT or R192Q KI mice were deeply anesthetized with i.p. urethane (0.3 ml of 1 g/ml; Sigma) and perfused transcardially with PBS followed by 4% paraformaldehyde. Trigeminal ganglia were removed, postfixed for 1 h at room temperature and cryoprotected overnight in 30% sucrose at 4°C. Each immunohystochemistry experiment was performed on an average of 5 cryostat-cut serial longitudinal slices (14 µm-thick) sampled every ∼70 µm, and thus covering the entire ganglion. Samples were incubated in a blocking solution containing 5% bovine serum albumin, 1% fetal bovine serum and 0.1% Triton X-100 in phosphate saline buffer for 2 h at room temperature, and immunostained with primary (for 16 h at 4°C) and secondary antibodies (2 h at room temperature). The following antibodies were used: anti-β-Tubulin III (1∶1000; Sigma); anti-Iba1 (1∶300; Wako, Richmond, VA, USA; [31], [32]); anti-TNFα antibody (1∶100, eBioscience, [46]); anti-biotin-F4/80 (1∶50; eBioscience, S.Diego, CA, USA; specificity of anti-F4/80 immunoreactivity is shown in Figure S2 [41], [42], [84]), anti-CD11b (1∶50; eBioscience; [85]); anti-ED-1 (1∶50; AbD Serotec, Oxford, UK; [86]); anti-glutamine synthetase (1∶150; Millipore, Milan, Italy; [33]), AlexaFluor488- or 594-conjugated antibodies (1∶500; Invitrogen, Milan, Italy). For F4/80 immunoreactivity, streptavidin-AlexaFluor 647 antibodies (1∶100, Invitrogen) were used. Nuclei were counterstained with DAPI (Sigma). Specificity of anti-Iba1 antibody was validated with Western immunoblotting (Figure S2) of extracts from WT and R192Q KI trigeminal lysates and purified macrophage population extracted from mouse peritoneum used as positive control [87]. Images from whole ganglion sections were visualized with Leica confocal microscope (Leica TCS SP2, Wetzlar, Germany) or a Zeiss Axioskop fluorescence microscope (Zurich, Switzerland). Cellular imaging and analysis of 3D reconstruction (Z-stack; 0.5 µm steps) of high magnification confocal images (Leica, Wetzlar, Germany) and co-expression analysis of multiple antigens were obtained with Volocity 5.5 software (Perkin Elmer, Waltham, MA, USA) and ImageJ Voxel counter (voxel, in µm^3^). Cell counting was carried out with MetaMorph software (Molecular Devices, Downingtown, PA, USA). The average total number of ganglion cells stained with DAPI was larger in R192Q KI than WT ganglia (1300±20 vs 1120±20, *n* = 4 mice). In view of the cell heterogeneity, in each experiment we compared equivalent regions of interest (ROIs) from WT and R192Q KI samples, processed and examined in parallel. Full details of ROIs are indicated in each Figure legend. Morphological and histological subdivision of neuronal-enriched V1, V2 and V3 areas of trigeminal ganglia is consistent with imaging experiments following retrograde labeling of trigeminal neurons [30], [37].

### Western blot

For western blotting, cells were lysed as previously detailed [10] in ODG buffer (2% n-octyl-beta-D-glucopyranoside, contaning 1% Nonidet P-40, 10 mM Tris pH 7.5, 150 mM NaCl plus protease inhibitors mixture; Complete, Roche Applied Science) and immunoblotted with rabbit anti-Iba1 antibodies (1∶1000, Wako), anti-F4-80 (1∶1000, eBioscience), anti-MCP-1 antibodies (1∶1000; Santa Cruz, CA, USA) or anti-actin antibodies (1∶3000, Sigma). Signals were detected with the enhanced chemiluminescence light system ECL (Amersham Biosciences, Piscataway, NJ, USA) and recorded by the digital imaging system Alliance 4.7 (UVITEC, Cambridge, UK). Quantification of the optical density of the bands was performed with ImageJ software plug-in.

### RNA isolation, reverse transcription and quantitative real-time-PCR

Total mRNA was extracted from intact ganglia as described before [10]. Single strand cDNA was obtained from 1 µg of purified RNA using the SuperScript III Reverse Transcriptase (Invitrogen) according to manufacture's instructions. Reactions were performed in duplicate in an iCycler IQ Real Time PCR System (Bio-Rad, Hercules, CA, USA) using IQ SyBr Green Supermix Reactions (Bio-Rad) and primers listed in Table 1. Control experiments have been performed following MIQE guidelines [88], confirming no significant difference in Ct between WT and R192Q KI ganglia extracts for GAPDH and β-tubulin III housekeeping expression (Ct_GAPDH_ WT 17.4±0.2, R192Q KI 17.34±0.06; Ct_β-tubulin III_ WT 17.5±0.4, R192Q KI 17.9±0.8). The relative mRNA expression in the different samples was normalized on housekeeping levels, namely glyceraldehyde 3-phosphate dehydrogenase (GAPDH) and β-tubulin III mRNA. Primary macrophages extracted from WT mice 72 h after a single intra-peritoneal injection of Brewer thioglycollate medium (0.4 g/kg, 3% wt/vol, Sigma, Milan, Italy) [87], were plated to Petri dishes and used for RNA extraction after 48 h.

**Table 1:** Real time PCR primers.

name	Primers	Locus	Length (bp)	Tm
GAPDH	Fw: 5′- AGAAGGTGGTGAAGCAGGCATC - 3′	NM_008084	111	60°C
	Rw: 5′- CGAAGGTGGAAGAGTGGGAGTTG - 3′			
β Tub III	Fw: 5′- CGCCTTTGGACACCTATTC - 3′	NM_023279	240	58°C
	Rw: 5′- TACTCCTCACGCACCTTG- 3′			
IL1β	Fw: 5′- TCTATACCTGTCCTGTGTAATGAAAG - 3′	NM_008361	195	55.5°C
	Rw: 5′- GGCTTGTGCTCTGCTTGTGAG - 3′			
IL6	Fw: 5′- GAGCCCACCAAGAACGATAGTC - 3′	NM_031168	96	60°C
	Rw: 5′- CCAGCATCAGTCCCAAGAAGG - 3′			
IL10	Fw: 5′- GGACTTTAAGGGTTACTTGGG - 3′	NT_078297	174	60°C
	Rw: 5′- AGAAATCGATGACAGCGCCT -3′			
TNFα	Fw: 5′- GTGGAACTGGCAGAAGAG - 3′	NM_013693	196	55.5°C
	Rw: 5′- CCATAGAACTGATGAGAGG -3′			
MCP-1	Fw: 5′- TTTTGTCACCAAGCTCAAGAGAG - 3′	NM_011333.3	248	60°C
	Rw: 5′- TCACTGTCACACTGGTCACTCC - 3′			
Cav2.1	Fw: 5′- GAAGTCCATCATAAGTCTGTTGTT - 3′	NM_007578	82	60°C
	Rw: 5′- GCCACCGAACAGCTGCAT - 3′			

In certain experiments, mRNA levels were expressed as fractional increase over saline-injected mice. Calculations for relative mRNA transcript levels were performed using the comparative method between cycle thresholds of different reactions as described before [88], [89], [90].

### ELISA analysis

Cytokines were quantified in lysates of trigeminal ganglia with a custom-made SearchLight Mouse Cytokine Array I (Aushon Biosystem, Billerica, MA, USA) and the Mouse TNFα ELISA assay (Thermo Scientific, Rockford, IL, USA). In each experiment, total protein content was evaluated with BCA kit (Sigma). Data have been normalized over the total protein content, in accordance with the manufacturer's instructions. Each sample was run at least in duplicate for each experiment.

### Statistical analysis

Data were collected from at least three independent experiments, and are expressed as mean ± standard error of the mean, where *n* indicates the number of independent experiments or the number of investigated cells, as indicated in the respective figure legend. Statistical analysis was performed using the Student's *t*-test or the Mann-Whitney rank sum test, depending on whether the data were normally distributed, or not (Sigma Plot and Systat Software Inc., San Jose, CA, USA). Multiple comparisons were analyzed with the One-way ANOVA and Tukey post-test.

## Supporting Information

Figure S1 **Iba1-CD11b colocalisation.** Representative confocal image of Iba1 (red) and CD11b (green) immunopositive cell from fiber-enriched areas from KI trigeminal ganglia highlight the co-localisation of these antigens. Nuclei were labeled with DAPI (blue). Scale bar: 3 µm.
(TIF)

Figure S2 **Specificity of antibodies.** Example of western immunoblots experiments of WT and KI trigeminal ganglia total protein lysates or peritoneal macrophages (MΦ), immunoprobed with antibodies against Iba1 (*A*) or F4/80 (*B*). Molecular weight of Iba1 and F4/80 were also indicated. Bottom lanes show total extracts equal loading levels, visualized with anti-actin antibodies.
(TIF)
