# Supplementary figures and images for: TNFα Levels and Macrophages Expression Reflect an Inflammatory Potential of Trigeminal Ganglia in a Mouse Model of Familial Hemiplegic Migraine
Source: PLoS One. 2013 Jan 11;8(1):e52394. doi: 10.1371/journal.pone.0052394 (PMC3543418; doi:10.1371/journal.pone.0052394)

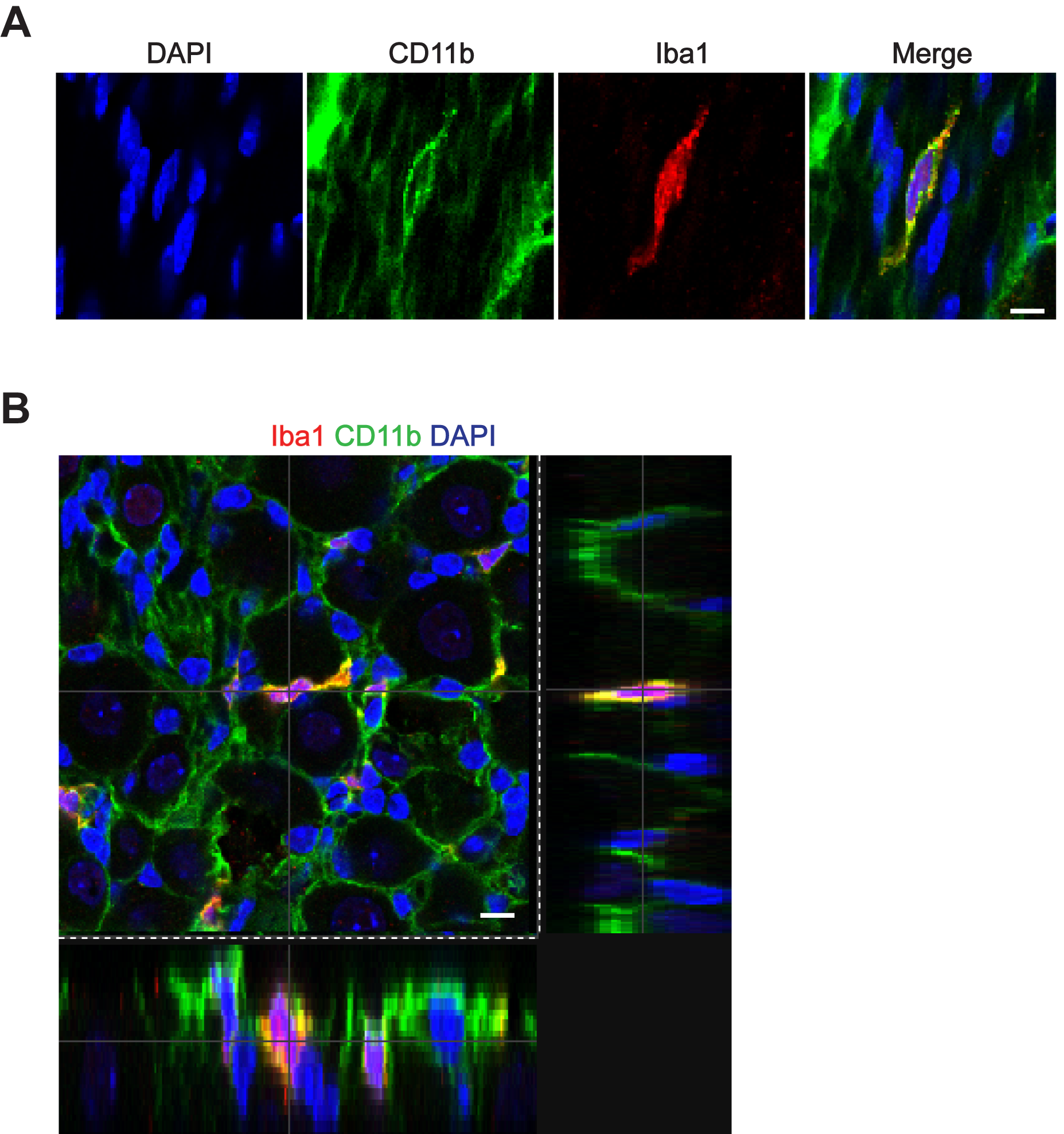

Supplement: Figure S1 — Iba1-CD11b colocalisation. Representative confocal image of Iba1 (red) and CD11b (green) immunopositive cell from fiber-enriched areas from KI trigeminal ganglia highlight the co-localisation of these antigens. Nuclei were labeled with DAPI (blue). Scale bar: 3 µm. (TIF) [file pone.0052394.s001.tif]

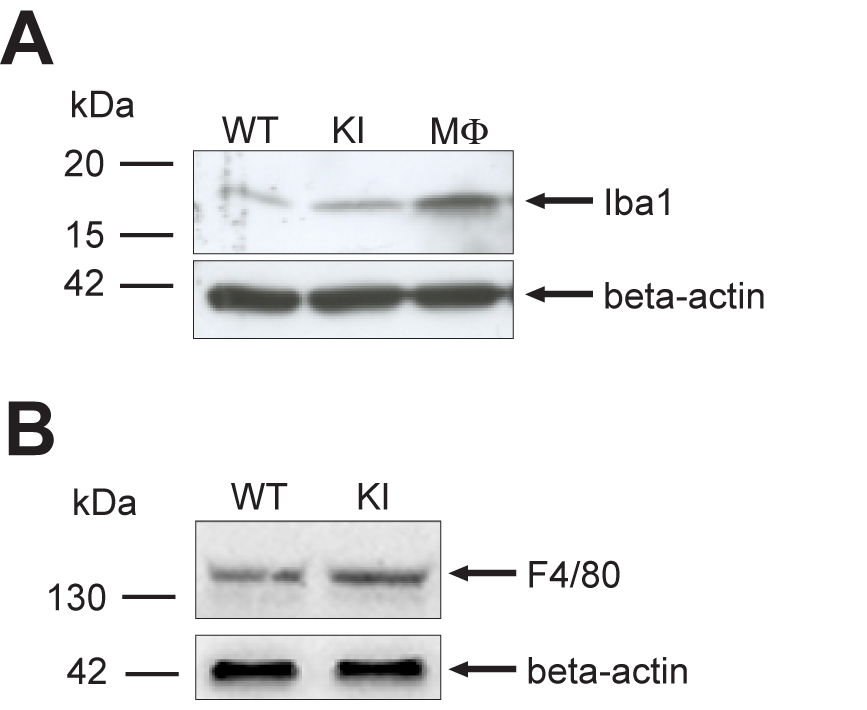

Supplement: Figure S2 — Specificity of antibodies. Example of western immunoblots experiments of WT and KI trigeminal ganglia total protein lysates or peritoneal macrophages (MΦ), immunoprobed with antibodies against Iba1 (A) or F4/80 (B). Molecular weight of Iba1 and F4/80 were also indicated. Bottom lanes show total extracts equal loading levels, visualized with anti-actin antibodies. (TIF) [file pone.0052394.s002.tif]
